# Acute Hypercortisolemia Exerts Depot-Specific Effects on Abdominal and Femoral Adipose Tissue Function

**DOI:** 10.1210/jc.2016-3600

**Published:** 2017-02-16

**Authors:** Konstantinos N. Manolopoulos, Michael W. O’Reilly, Iwona J. Bujalska, Jeremy W. Tomlinson, Wiebke Arlt

**Affiliations:** 1 Institute of Metabolism and Systems Research, University of Birmingham B15 2TT, United Kingdom; 2 Centre for Endocrinology, Diabetes and Metabolism, Birmingham Health Partners, Birmingham B15 2TH, United Kingdom; 3 Oxford Centre for Diabetes, Endocrinology and Metabolism, National Institute for Health Research Oxford Biomedical Research Centre, University of Oxford, Churchill Hospital, Oxford OX3 7LJ, United Kingdom; 4 National Institute for Health Research Birmingham Liver Biomedical Research Unit, University Hospitals Birmingham, National Health Service Foundation Trust, Birmingham B15 2TH, United Kingdom

## Abstract

**Context:**

Glucocorticoids have pleiotropic metabolic functions, and acute glucocorticoid excess affects fatty acid metabolism, increasing systemic lipolysis. Whether glucocorticoids exert adipose tissue depot-specific effects remains unclear.

**Objective:**

To provide an *in vivo* assessment of femoral and abdominal adipose tissue responses to acute glucocorticoid administration.

**Design and Outcome Measures:**

Nine healthy male volunteers were studied on two occasions, after a hydrocortisone infusion (0.2 mg/kg/min for 14 hours) and a saline infusion, respectively, given in randomized double-blind order. The subjects were studied in the fasting state and after a 75-g glucose drink with an *in vivo* assessment of femoral adipose tissue blood flow (ATBF) using radioactive xenon washout and of lipolysis and glucose uptake using the arteriovenous difference technique. In a separate study (same infusion design), eight additional healthy male subjects underwent assessment of fasting abdominal ATBF and lipolysis only. Lipolysis was assessed as the net release of nonesterified fatty acids (NEFAs) from femoral and abdominal subcutaneous adipose tissue.

**Results:**

Acute hypercortisolemia significantly increased basal and postprandial ATBF in femoral adipose tissue, but the femoral net NEFA release did not change. In abdominal adipose tissue, hypercortisolemia induced substantial increases in basal ATBF and NEFA release.

**Conclusions:**

Acute hypercortisolemia induces differential lipolysis and ATBF responses in abdominal and femoral adipose tissue, suggesting depot-specific glucocorticoid effects. Abdominal, but not femoral, adipose tissue contributes to the hypercortisolemia-induced systemic NEFA increase, with likely contributions from other adipose tissue sources and intravascular triglyceride hydrolysis.

## Teaser

Acute experimental hypercortisolemia induces differential metabolic responses in abdominal and femoral adipose tissue in vivo, suggesting depot-specific glucocorticoid effects in humans.

Glucocorticoids (GCs) are important pleiotropic hormones that exert anabolic effects during physiological conditions but also play a pivotal role in tissue catabolism when acutely elevated as part of an acute stress response (1). In contrast, chronic pathophysiological GC excess in Cushing syndrome is characterized by a distinctive centripetal fat mass redistribution, in particular, visceral fat mass accumulation, and is associated with increased morbidity and mortality (2, 3). The body fat mass distribution is an important determinant of health, and abdominal fat mass accumulation represents a major risk factor for cardiovascular disease (4). In contrast, a larger thigh subcutaneous fat mass has been independently associated with favorable cardiovascular and metabolic profiles (5), and the relative scarcity of femoral fat might have important implications for cardiometabolic health (6, 7). The femoral adipose tissue mass is determined by the balance between fatty acid uptake and their release, lipolysis, with the latter regulated *in vivo* in a depot-specific manner via selective adrenoceptor action (8).

Acute hypercortisolemia results in an increase of whole body lipolysis (*i.e.,* the release of nonesterified fatty acids [NEFAs] from adipose tissue); however, the exact effects of GCs on depot-specific lipolysis remain unclear (9). Murine *in vitro* models have yielded conflicting results, with only some reporting induction of lipolysis by GCs in a dose-dependent manner [reviewed by Lee *et al.* (1)]. In intact human adipocytes isolated from the abdominal subcutaneous depot, cortisol treatment resulted in inhibition of basal and *β*-adrenergic–mediated lipolysis (10). In contrast, lipolysis appeared unaffected by the synthetic GC dexamethasone (11, 12). Early *in vivo* studies also showed that GCs inhibit sympathetic activity and adrenoceptor function in humans (13, 14), suggesting this could affect lipolytic responses. However, femoral lipolysis, determined as glycerol release using the microdialysis technique, has been found to either increase (15) or remain unchanged (16) in the presence of experimental GC excess.

The aim of our study was to examine lipolysis in response to acute GC excess, using an integrative *in vivo* physiology approach. In particular, we sought to investigate the depot-specific contribution of femoral adipose tissue to whole body lipolysis during hypercortisolemia. Based on previous studies (17, 18), we hypothesized that acute elevation of cortisol concentrations would affect femoral adipose tissue blood flow (ATBF) and NEFA release, in line with an acute catabolic response. Furthermore, we studied the femoral adipose tissue postprandial glucose uptake and, in a separate study, abdominal adipose tissue fasting ATBF and NEFA release as markers of depot-specific adipose tissue function *in vivo*.

## Methods

### Subjects

Healthy male individuals with no medical condition and not receiving any drug therapy were recruited using print and electronic advertising. All subjects underwent a medical evaluation during the screening visit to ensure they were healthy and had normal liver and kidney function parameters and normal blood counts. No subject had any relevant medical history, smoked tobacco, or took any regular medications that could affect the study’s outcome measures. All parts of the present study were conducted at the National Institute for Health Research/Wellcome Trust Clinical Research Facility of the University of Birmingham/Queen Elizabeth Hospital Birmingham. The Solihull National Health System Research Ethics Committee approved the present study (approval no. 12/WM/0327), and all subjects gave informed consent in writing before participation.

### Study design

#### Anthropometric measurements

Measurements were taken during the screening visit. The waist circumference was measured midway between the lower margin of the last palpable rib and the top of the iliac crest, and the hip circumference was measured at the level of the greater trochanters. The total and regional fat mass (including the estimated visceral fat mass) were measured using dual-energy x-ray absorptiometry (19). The blood pressure and heart rate were measured using a standard oscillometric blood pressure monitor with an upper arm cuff.

#### Study visits

**Figure 1.**
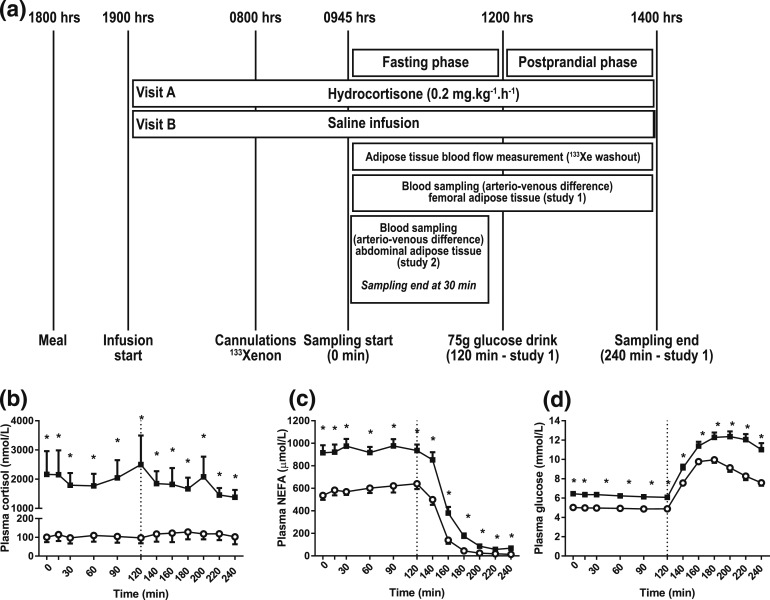
(a) Study design and systemic metabolite concentrations. Healthy male volunteers underwent the study twice, receiving either a constant overnight hydrocortisone or saline infusion until the end of the study. Infusions were given in a randomized, double-blind order. A standard 75-g glucose drink was given at 120 minutes (n = 9; study 1). In 8 further individuals (study 2), blood sampling across abdominal adipose tissue was performed in the fasting state only. (b) Plasma cortisol, (c) NEFA, and (d) glucose during hydrocortisone (black squares) or saline (open circles) infusion. Hypercortisolemia increased plasma NEFA and glucose concentrations. Data from study 1, n = 9; *P < 0.05 compared with saline for each time point, Wilcoxon test.

For each of the two study visits, the subjects were admitted to the National Institute for Health Research/Wellcome Trust Clinical Research Facility at 5 pm, and a cannula for infusion purposes was inserted into an antecubital fossa vein [Fig. 1(a)]. At 6 pm, they were served a standardized calorie-controlled meal (vegetable lasagna; energy, 2634 kJ; total nutritional content, 14.4 g fat, 93 g carbohydrates, 25.2 g protein, 11.4 g fiber). They then fasted until study completion the next day. At 7 pm, a constant infusion of either hydrocortisone (0.2 mg/kg/h, total dose infused equivalent to the doses commonly used in the setting of adrenal insufficiency and acute illness) or saline (control study visit) was started and continued for 14 hours until study completion the next day. The 2 study days were separated by ≥2 weeks, and the hydrocortisone and saline infusions were administered in a double-blind, randomized fashion. At 10 pm, the lights were switched off for night rest. At 8:00 am the next morning, catheters were placed into veins draining the femoral (study 1) or abdominal (study 2) subcutaneous adipose tissue (see later) (20, 21). A further cannula was inserted either retrogradely into a vein of the dorsal hand, which was then placed in a hot box at 60°C to obtain arterialized blood samples (study 1), or in a radial artery (study 2). For the ATBF measurement, ^133^Xe was injected into femoral (study 1) or abdominal (study 2) subcutaneous adipose tissue (22), and scintillation was measured continuously using customized cesium detectors (GMS411 System; John Caunt Scientific, Bury, UK). Subsequently, study participants were left to relax for 45 minutes to allow equilibration of ^133^Xe. Before the start of blood sampling, the blood pressure and heart rate were measured. After study completion, all catheters were removed, and the subjects were given a full meal.

#### Region-specific blood sampling

In 9 participants (study 1), a venous catheter was placed into the femoral saphenous vein. After blood samples taken at regular intervals for 2 hours (fasting phase), a standardized 75-g glucose drink was given at 120 minutes. Blood sampling continued for another 2 hours. In a separate study (study 2) with the same infusion design and duration as outlined, we studied abdominal ATBF and NEFA release in eight participants by taking samples from a superficial epigastric vein, with sampling performed under fasting conditions only. In both studies, all venous and arterial and arterialized samples were taken simultaneously at specified times (arteriovenous difference technique) (20, 23).

### Analytical methods

The blood samples were drawn into heparinized syringes, and plasma was prepared rapidly at 4°C and immediately frozen at −80°C before analysis. The plasma glucose, NEFA, and glycerol concentrations were measured enzymatically using commercially available kits on an ILAB600 or ILAB650 clinical analyzer (Instrumentation Laboratory UK, Warrington, UK). Cortisol was measured using a colorimetric assay (R&D Systems, Abingdon, UK). Insulin and C-peptide were measured using an enzyme-linked immunosorbent assay (Invitron, Monmouth, UK) in an accredited reference laboratory (Diabetes Research Unit, Swansea University, Swansea, UK). Interleukin (IL)-6 was measured using an enzyme-linked immunosorbent assay (Thermo Fisher Scientific, Loughborough, UK).

### Calculations and statistical analysis

Indexes of pancreatic *β*-cell function and insulin resistance were calculated using the updated computer model-based homeostatic model assessment (HOMA) method (24); the calculations are given in the Supplemental Methods section. The mean of three consecutive plasma glucose and insulin fasting measurements (time points, 0 to 30 minutes) was used for HOMA calculations. Metabolite uptake and release across abdominal and femoral adipose tissue were calculated using the arteriovenous difference technique (8). For NEFA and glycerol release, the venoarterial concentration difference was multiplied by the ATBF for each of the abdominal and femoral adipose tissue depots, respectively. For glucose uptake, the arteriovenous concentration difference was multiplied by the ATBF. A detailed description of all calculations is given in the Supplemental Methods section. For study 1, comparisons between control and hypercortisolemia were performed for each time point (pairwise comparisons) or by comparing areas under the curve (AUCs). The AUC was also used for comparisons between the fasting and postprandial states. The AUC was calculated using the trapezoid rule and is presented as a time-averaged value (AUC divided by the relevant time period). For study 2, the mean of three fasting measurements (time points, 0 to 30 minutes) was used for all calculations and comparisons. To calculate the ratios and re-esterification rates in studies 1 and 2, the mean of three measurements (time points, 0 to 30 minutes) was used to calculate the fasting data. For the femoral adipose tissue, the mean of time points 200 to 240 minutes was used for the postprandial calculations. The data distribution was tested for normality using the Shapiro-Wilk test, and parametric or nonparametric tests were used, as indicated. Data were analyzed using IBM Statistics for Windows, version 21 (Armonk, NY), and GraphPad Prism for Windows, version 6.05 (San Diego, CA). *P* < 0.05 was considered statistically significant. All data are presented as mean ± standard error of the mean, unless otherwise stated.

## Results

The baseline anthropometric and metabolic characteristics of the subjects are listed in Table 1. Hydrocortisone infusion resulted in a mild increase in the heart rate (*P* = 0.033 for study 1, *P* = 0.022 for study 2, compared with the control; paired *t* test) but did not affect the blood pressure.

**Table 1.** **Baseline Anthropometric and Metabolic Characteristics of Participants**

**Characteristic**	**Study 1 (n = 9)**	**Study 2 (n = 8)**	***P* Value**
Age, y	28 (19-57)	23 (19-47)	0.53
Weight, kg	87.3 (67.3-96.4)	80.2 (74.0-93.0)	0.37
BMI, kg/m^2^	26.5 (22.7-30.4)	24.1 (23.1-27.5)	0.13
Waist/hip ratio	0.9 (0.76-1.03)	0.87 (0.78-0.98)	0.47
Trunk fat mass, kg	13.5 (5.2-19.8)	9.0 (5.9-15.2)	0.24
Leg fat mass, kg	6.4 (2.9-12.4)	6.1 (4.6-12.4)	0.77
Visceral fat mass, kg	0.7 (0.3-1.7)	0.3 (0.2-1.2)	0.16
BP systolic, mm Hg			
Control	129 ± 5	131 ± 3	0.68
Hypercortisolemia	129 ± 6	128 ± 3	0.91
BP diastolic (mm Hg)			
Control	74 ± 3	70 ± 2	0.07
Hypercortisolemia	73 ± 4	67 ± 3	0.26
Heart rate (bpm)			
Control	62 ± 4	63 ± 3	0.72
Hypercortisolemia	73 ± 5*^a^*	73 ± 3*^b^*	1.0

Data presented as median (range) or mean ± standard error of the mean.

Abbreviations: BMI, body mass index; BP, blood pressure.

^a^ *P* = 0.033 compared with control.

^b^ *P* = 0.020 compared with control.

### Hydrocortisone infusion led to increased systemic NEFA and peripheral insulin resistance

In study 1 (femoral adipose tissue depot), hydrocortisone infusion resulted in a substantial increase in cortisol concentrations [AUC, 0 to 240 minutes, saline *vs* hydrocortisone, *P* = 0.008, Wilcoxon test; Fig. 1(b)]. Hypercortisolemia had substantial effects on plasma NEFA and glucose concentrations. Compared with the control, the plasma NEFA increased 1.6-fold in the fasting state (AUC, 0 to 120 minutes, saline *vs* hydrocortisone, *P* = 0.008, Wilcoxon test). After glucose ingestion, the plasma NEFA concentrations became suppressed under control conditions (AUC, 0 to 120 minutes *vs* AUC, 120 to 240 minutes, *P* = 0.008, Wilcoxon test). In hypercortisolemia, the plasma NEFA concentrations also became suppressed after glucose ingestion (AUC, 0 to 120 minutes *vs* AUC, 120 to 240 minutes, *P* = 0.008, Wilcoxon test). Compared with the control, the postprandial plasma NEFA concentrations remained higher during hydrocortisone infusion [AUC, 120 to 240 minutes, saline *vs* hydrocortisone, *P* = 0.008, Wilcoxon test; Fig. 1(c)]. In response to hydrocortisone infusion, the plasma glucose increased 1.3-fold in both the fasting state (AUC, 0 to 120 minutes, saline *vs* hydrocortisone, *P* = 0.008, Wilcoxon test) and the postprandial state (AUC, 120 to 240 minutes, saline *vs* hydrocortisone, *P* = 0.008, Wilcoxon test) compared with control study visits [Fig. 1(d)].

**Figure 2.**
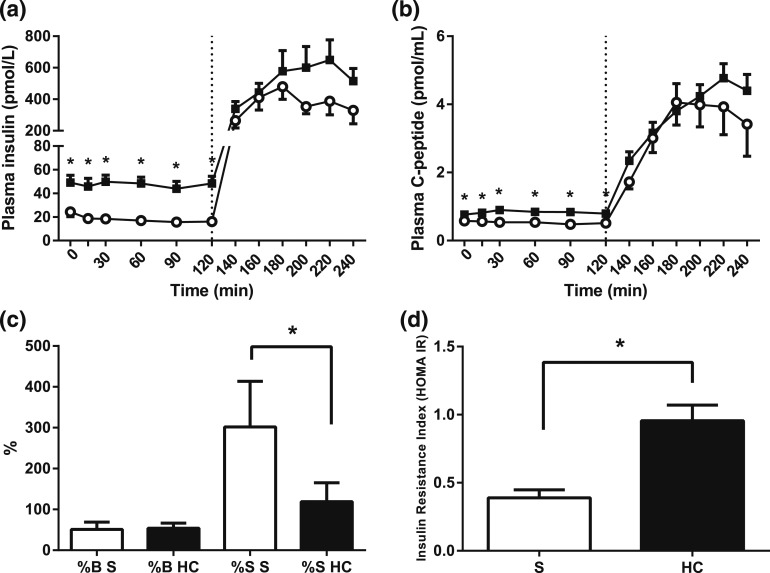
Pancreatic function and insulin resistance indexes (HOMA). (a) Plasma insulin and (b) C-peptide concentrations during hydrocortisone (HC; black squares) or saline infusion (S; open circles). A standard 75-g glucose drink was given at 120 minutes. HOMA indexes of (c) pancreatic *β*-cell function (%B) and insulin sensitivity (%S) and (d) insulin resistance index (IR). Hypercortisolemia increased basal insulin and C-peptide concentrations and resulted in peripheral insulin resistance. Data from study 1, n = 9; *P < 0.05 compared with saline for each time point, Wilcoxon test.

Overall, the plasma insulin concentrations increased during hydrocortisone infusion [AUC, 0 to 240 minutes, saline *vs* hydrocortisone, *P* = 0.018, Wilcoxon test; Fig. 2(a)]. This was more pronounced for fasting (*i.e.,* basal, insulin secretion; individual time points, 0 to 120 minutes, saline *vs* hydrocortisone, *P* < 0.001 for infusion type, analysis of variance [ANOVA]). In contrast, glucose-stimulated secretion, in terms of both response time and magnitude, appeared less affected by hydrocortisone (individual time points, 120 to 240 minutes, saline *vs* hydrocortisone, *P* = 0.051 for infusion type; *P* = 0.077 for infusion type × time; ANOVA). Plasma C-peptide secretion responses to hydrocortisone infusion mirrored that of insulin, with a relevant fasting increase compared with control (individual time points, 0 to 120 minutes, saline *vs* hydrocortisone, *P* < 0.001 for infusion type, ANOVA). Similarly to insulin, this increase was not seen in the postprandial phase [individual time points, 120 to 240 minutes, saline *vs* hydrocortisone, *P* = 0.441 for infusion type, *P* = 0.093 for infusion type × time, ANOVA; Fig. 2(b)]. Accordingly, pancreatic *β*-cell function did not change significantly during hydrocortisone infusion compared with control (saline *vs* hydrocortisone, *P* = 0.374, Wilcoxon test), but insulin sensitivity did decrease dramatically [saline *vs* hydrocortisone, *P* = 0.008, Wilcoxon test; Fig. 2(c)]. In line with this, insulin resistance increased during hydrocortisone infusion [saline *vs* hydrocortisone, *P* = 0.012, Wilcoxon test; Fig. 2(d)].

### Fasting and postprandial femoral ATBF increased during hypercortisolemia

**Figure 3.**
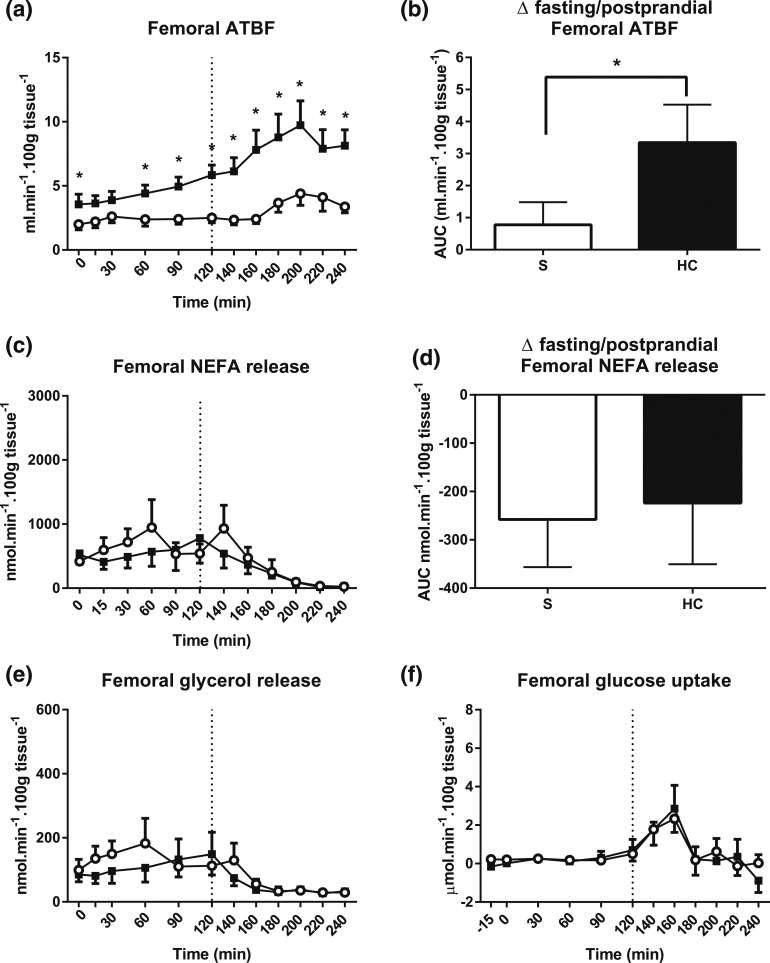
Femoral adipose tissue responses. (a) Femoral ATBF during hydrocortisone (HC, black squares and bars) or saline (S, open circles and bars) infusion. A standard 75-g glucose drink was given at 120 minutes. (b) ATBF change in the fasting to postprandial state transition. (c) Femoral NEFA release and (d) lipolysis suppression induced by glucose ingestion. (e) Femoral glycerol release and (f) glucose uptake. Hypercortisolemia markedly increased basal and postprandial ATBF but did not have any effect on femoral lipolysis or postprandial femoral glucose uptake. Release was calculated by multiplying the venoarterial concentration difference with ATBF. Uptake was calculated by multiplying the arteriovenous concentration difference with ATBF. Data from study 1, n = 9; *P < 0.05 compared with saline for each time point, Wilcoxon test.

During control conditions, femoral ATBF was stable in the fasting phase and after glucose ingestion [individual time points, 0 to 240 minutes, saline, *P* = 0.155 prandial state × time, ANOVA; Fig. 3(a)]. Hydrocortisone infusion induced a steady increase in femoral ATBF during the fasting phase (individual time points, 0 to 120 minutes, saline *vs* hydrocortisone, *P* = 0.009 infusion type × time, ANOVA). After glucose ingestion, a further marked increase occurred in femoral ATBF compared with control (AUC, 120 to 240 minutes, saline *vs* hydrocortisone, *P* = 0.011, Wilcoxon test). In addition, hydrocortisone induced a larger ATBF change in the fasting to postprandial state transition [ΔAUC fasting/postprandial saline *vs* hydrocortisone, *P* = 0.024, Wilcoxon test; Fig. 3(b)].

### Femoral lipolysis and glucose uptake remained unaffected by hypercortisolemia

The fasting femoral NEFA release rate was 671 ± 232 nmol/min/100 g tissue (AUC, 0 to 120 minutes) under control conditions [Fig. 3(c)]. Expectedly, after glucose ingestion, lipolysis was inhibited (AUC, 0 to 120 minutes *vs* 120 to 240 minutes, *P* = 0.021, Wilcoxon test). During hydrocortisone infusion, fasting and postprandial NEFA release rate were similar to control conditions [individual time points, 0 to 120 minutes, *P* = 0.285; and individual time points, 120 to 240 minutes, *P* = 0.447, infusion type × time, respectively; ANOVA, saline *vs* hydrocortisone; Fig. 3(c)]. No difference was found in the degree of lipolysis suppression by glucose [ΔAUC, fasting/postprandial for saline *vs* hydrocortisone, *P* = 0.824, Wilcoxon test; Fig. 3(d)]. Regional glycerol release, a further marker of lipolysis, essentially mirrored NEFA release [Fig. 3(e)].

During control conditions, the fasting femoral adipose tissue glucose uptake rate was 0.2 ± 0.1 µmol/min/100 g tissue [AUC, 0 to 120 minutes; Fig. 3(f)]. After glucose ingestion, uptake had peaked to 2.3 ± 0.7 µmol/min/100 g tissue at 160 minutes. However, the overall postprandial AUC remained unchanged compared with that at fasting (AUC, 0 to 120 minutes *vs* AUC, 120 to 240 minutes, *P* = 0.102, Wilcoxon test). The fasting and postprandial glucose uptake responses remained virtually the same during both control and hydrocortisone infusion states (AUC, 0 to 240 minutes, saline *vs* hydrocortisone, *P* = 0.926, depot × prandial state × infusion type, ANOVA).

### Acute hypercortisolemia increased fasting abdominal ATBF and lipolytic rate

**Figure 4.**
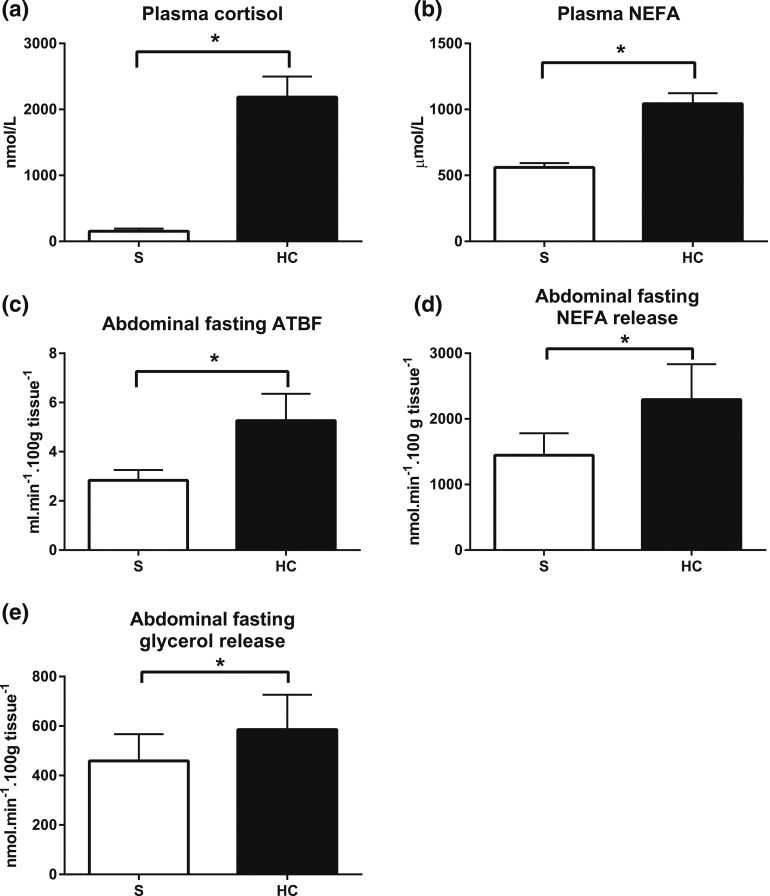
Abdominal fasting ATBF and lipolysis. (a) Plasma cortisol and (b) fasting NEFA concentrations during hydrocortisone (HC, black bars) or saline (S, white bars) infusion. (c) Abdominal fasting ATBF, (d) NEFA release, and (e) glycerol release during control and hydrocortisone infusions. Hypercortisolemia increased abdominal ATBF and NEFA release. Bars represent the mean of three fasting measurements (time points, 0 to 30 minutes). Release was calculated by multiplying the venoarterial concentration difference with ATBF. Uptake was calculated by multiplying the arteriovenous concentration difference with ATBF. Data from study 2, n = 8; *P < 0.05, paired *t* test.

In study 2 (abdominal adipose tissue depot), we compared fasting abdominal ATBF and lipolysis in the presence of hypercortisolemia to control conditions using the same infusion regimen. This again resulted in substantial increases in plasma cortisol [Fig. 4(a)] and NEFA [Fig. 4(b)] concentrations. Given that the study was performed in the fasting state only, we did not measure the abdominal adipose tissue glucose uptake. Hypercortisolemia increased the fasting ATBF [mean, 0 to 30 minutes, saline *vs* hypercortisolemia, *P* = 0.039, paired *t* test; Fig. 4(c)]. The abdominal fasting net NEFA release also increased compared with that of the control [mean, 0 to 30 minutes, saline, 1446 ± 334 nmol/min/100 g tissue *vs* hypercortisolemia, 2293 ± 541 nmol/min/100 g tissue, *P* = 0.031, paired *t* test; Fig. 4(d)], as did the abdominal fasting net glycerol release [mean, 0 to 30 minutes, saline *vs* hypercortisolemia, *P* = 0.021, paired *t* test; Fig. 4(e)].

### NEFA handling in femoral and abdominal adipose tissue

Because of the increased systemic NEFA concentrations and the marked increases in ATBF observed during hypercortisolemia, we sought to calculate a marker of NEFA handling in each depot that is independent of ATBF. Thus, as a marker of local lipolysis at the adipocyte level, we calculated the fasting venoarterial NEFA ratio. In femoral adipose tissue, the ratio decreased from 1.8 ± 0.2 under control conditions to 1.3 ± 0.1 in hypercortisolemia (mean, 0 to 30 minutes, saline *vs* hydrocortisone, *P* = 0.035, Wilcoxon test). Similarly, in the abdominal adipose tissue, it decreased from 2.3 ± 0.2 to 1.7 ± 0.1 (mean, 0 to 30 minutes, *P* = 0.035, Wilcoxon test).

### Systemic and regional adipose tissue IL-6 production

Cortisol exerts a wide range of nonmetabolic effects, and we measured IL-6 production as an inflammatory marker in response to the hydrocortisone infusion (Supplemental Fig. 1). The systemic fasting plasma IL-6 concentrations did not change significantly between the control and hypercortisolemia conditions (time point, 0 minutes, 11.7 ± 3.6 pg/mL *vs* 12.7 ± 6.9 pg/mL; *P* = 0.798). Abdominal adipose tissue appeared to be a net producer of IL-6 (release control, 12.6 ± 5.6 pg/mL/100 g tissue; hypercortisolemia, 19.7 ± 9.7 pg/mL/100 g tissue). However this did not reach statistical significance (control, *P* = 0.109; hypercortisolemia, *P* = 0.087, one-sample *t* test compared with zero). Statistically, femoral adipose tissue IL-6 release was not different from zero (femoral IL-6 release, control, 1.14 ± 0.56 pg/mL/100 g tissue; hypercortisolemia, −0.28 ± 2.86 pg/mL/100 g tissue; *P* = 0.077 and *P* = 0.925, respectively; one-sample *t* test compared with zero).

## Discussion

GCs have important metabolic functions that are tissue specific and vary depending on the length of exposure (1). In relation to their effect on *in vivo* fatty acid metabolism, general agreement has been reached that hypercortisolemia increases the plasma NEFA concentrations (15, 17, 25, 26). However, it remains unresolved which adipose tissue depots contribute to this increase, and whether differential contribution of specific adipose tissue depots results in the phenotypic fat mass distribution changes observed with chronic GC excess. The exact source of NEFA is still debated, with some studies reporting an increase in abdominal lipolysis (15, 27), and others finding a reduction (17). Given the previously reported differences in the depot-specific regulation of lipolysis (8), we studied the effects of acute hypercortisolemia on the femoral and abdominal adipose tissue depot using an integrative *in vivo* physiology approach. We found that during hypercortisolemia, the abdominal net NEFA output increased, and femoral NEFA release remained stable, despite a striking increase in femoral ATBF. We have described the potential effects of the increased ATBF on local NEFA handling during hypercortisolemia and suggest that other depots (*e.g.,* visceral adipose tissue) might play a role in systemic NEFA concentration increases, in addition to the contribution of abdominal NEFA release.

Similar to previous studies (15, 17, 25, 26), we found a marked increase of systemic NEFA concentrations in the fasting state during GC infusion. Using isotope tracers, it has been shown that the palmitate and glycerol rates of appearance increase with hydrocortisone, suggesting induction of lipolysis at the systemic level (15, 17). Subcutaneous adipose tissue is the major source of NEFA, driven by the activity of adipocyte-specific lipases, most notably adipose tissue triglyceride lipase and hormone-sensitive lipase (28). The NEFA rate of appearance in the systemic circulation is determined by the ATBF and the net lipolytic rate of the individual adipose tissue depots (20, 29). We studied these parameters to measure the individual contribution of the femoral and abdominal depots to the increased systemic NEFA concentrations in hypercortisolemia, hypothesizing that femoral adipose tissue is a net contributor.

We found a steady increase in femoral ATBF in fasting conditions during GC infusion, with a further major increase in the postprandial phase. This is in stark contrast to the previously reported finding of a mostly unresponsive femoral ATBF (8). In contrast to our hypothesis, we found no differences in the femoral lipolytic rate during hydrocortisone infusion. Our data suggest a strong inhibition of lipolysis at the actual adipocyte level, because the net femoral lipolytic rate did not change after GC infusion, despite the marked local ATBF increase. We found a moderate increase in basal abdominal ATBF and a small net increase in fasting abdominal NEFA release during hypercortisolemia.

We did not measure the triglyceride concentrations, because we were not expecting any changes owing to the nature of the experimental meal stimulus (pure glucose); therefore, we could not calculate the exact transcapillary flux of fatty acids (30). Hence, the exact effects of GC excess on postprandial fatty acid trafficking would need to be studied in future experiments, ideally involving a lipid-containing meal.

ATBF is an important determinant of adipose tissue function, because it determines the influx and efflux of metabolites, systemic hormones, and adipokines (31). Basal ATBF is determined largely by nitric oxide, and the postprandial increase is mediated by *β*-adrenergic stimulation (32). Femoral ATBF is much less responsive to adrenergic stimulation owing to the prevailing inhibitory *α*2-adrenoceptor activity, underlining the concept of a depot-specific regulation (8). Vascular smooth muscle cell tone and endothelial nitric oxide synthesis are known to be affected by GC [reviewed by Yhang and Zhang (33)]. Although GCs inhibit endothelial nitric oxide synthase, they also inhibit sympathetic nerve outflow in humans (13, 14). In line with the latter, studies of humans have indicated that the resting systemic catecholamine rate of appearance does not change during hydrocortisone infusion and the effects are mediated at the level of end-organ responsiveness (34). However, GC-mediated inhibition of the prevailing *α*2-adrenoceptor signal in femoral adipose tissue could be a possible mechanism for the depot-specific effect on femoral ATBF observed in our studies.

In our studies of healthy volunteers, hydrocortisone infusion resulted in a substantial plasma cortisol increase, with abolition of the diurnal cortisol variation. Hypercortisolemia is known to induce insulin resistance (35) by affecting postreceptor insulin signaling and decreasing glucose clearance (36, 37). Our systemic metabolite data suggest that we were able to replicate the effects of GC excess, reflected by our findings of increased basal glucose and insulin and increased postprandial glucose concentrations. C-peptide secretion was preserved during hypercortisolemia, suggesting pancreatic *β*-cell function was maintained. In line with this, the HOMA indexes showed an increase in insulin resistance with unaltered *β*-cell function. Considering the high HOMA insulin sensitivity values found during control conditions, these data suggest that our study population consisted of very insulin-sensitive individuals who were rendered insulin resistant by the hydrocortisone infusion.

Previous findings have suggested the GC effects on insulin sensitivity are tissue specific, such that they might induce insulin resistance in muscle but increase insulin sensitivity in subcutaneous adipose tissue *in vivo* (26). Muscle is the main site of postprandial glucose uptake, and adipose tissue accounts only for a small proportion of glucose clearance (38). We found that adipose tissue-specific glucose uptake rates were not affected by GC-induced whole body insulin resistance, supporting an adipose tissue-specific insulin-sensitizing GC effect. Furthermore, insulin is the major inhibitor of adipose tissue lipolysis (28). In our study, glucose-mediated suppression of lipolysis was not affected by hydrocortisone, underlining the exquisite ability of insulin to maximally suppress hormone-sensitive lipase activity and providing further support that GCs do not induce adipose tissue insulin resistance (26).

To evaluate the nonmetabolic effects of the induced hypercortisolemia, we measured the IL-6 concentrations as one of the main inflammatory cytokines (39). The plasma concentrations of IL-6 increase as part of the acute inflammatory response pathway, and acute hydrocortisone administration is known to reduce IL-6 concentrations, although it appears that this effect only becomes apparent after a few days (40). Therefore, and because our study participants were healthy volunteers without any pathophysiological elevations in IL-6 concentrations, we did not see any effects of the hydrocortisone infusion on the systemic IL-6 concentrations. Previously, it has been shown that abdominal adipose tissue is a net producer of IL-6, and femoral adipose tissue is not (41). Our results appear to be in line with this, although they did not reach statistical significance, most likely owing to the small sample size.

A particular strength of our study was the integrative *in vivo* physiology approach chosen. We used arteriovenous differences and ATBF measurements for the assessment of NEFA release, the direct product of lipolysis, in response to GC excess under near-normal conditions. Most previous studies investigating adipose tissue depot-specific GC effects on lipolysis used the microdialysis technique, which relies on the measurement of interstitial glycerol concentrations as an indirect index of lipolysis (42). Only a few studies to date have included femoral adipose tissue responses (15, 16, 27). Djurhuus *et al.* (15, 27) found an increase in abdominal and femoral adipose tissue interstitial glycerol after short-term (6-hour) hydrocortisone infusion. When assessing metabolite fluxes, it is important to consider the depot-specific changes in blood flow. We showed that the marked femoral-specific ATBF effect in response to hypercortisolemia was augmented in the postprandial phase, mostly in response to insulin. Although insulin does not exert direct ATBF effects, it is known to be in important mediator (43). Also, in contrast to previous studies using acute GC excess as an experimental approach (15, 27), we saw a small, but important, increase in the basal plasma insulin concentrations. In previous studies, femoral ATBF was not measured; instead, abdominal ATBF was used as a basis for calculating femoral interstitial glycerol release (15, 16, 27). In these studies, abdominal ATBF did not change in response to hypercortisolemia. Because of the previously unknown marked femoral-specific ATBF effects we have described, it is possible that previous microdialysis studies overestimated femoral glycerol appearance. Our abdominal depot findings are in contrast to previous results from the only study using the same experimental approach we used. They found a substantial decrease in abdominal NEFA efflux during hydrocortisone infusion (17). This difference in findings could have resulted from the slightly higher plasma cortisol concentrations achieved in our study, resulting in an overall increased systemic fatty acid turnover, or other factors intrinsic to our study population such as differences in local 11*β*-HSD1 activity or ATBF responses.

Our study had some limitations by design, including the small size of our sample and that conclusions can be drawn only about men. The hydrocortisone infusion was calculated to provide approximately 300 mg/24 hours, a dose regularly used in clinical practice. This resulted in supraphysiological plasma cortisol concentrations, approximately double that found in acute stress situations (*e.g.,* sepsis) (44). To the best of our knowledge, no reported studies have examined the exact dose-dependent metabolic response in acute hypercortisolemia *in vivo.* Although acute dose-dependent GC effects cannot be excluded, it has been generally accepted that the tissue responses are more dependent on GR expression and local GC activity modulation by the 11*β*-HSD enzymes than absolute plasma concentrations (45). In support of this, our systemic metabolite data are comparable with those from studies that achieved more physiological hypercortisolemia (25). The acute nature of the hypercortisolemia limits the conclusions we can draw regarding depot-specific lipolysis in conditions of chronic GC excess (*i.e.,* Cushing syndrome). However, the finding of isolated fasting and postprandial ATBF induction in the femoral adipose tissue suggests an early signal of GC induced changes in this depot. Our calculations were performed based on the measurement of nonlabeled NEFA and glycerol only. Also, because our study protocol did not include any meal containing lipids, exact modeling of depot-specific fatty acid trafficking, in particular, triglyceride metabolism in the postprandial state, was not possible. Also, the metabolic flux calculations were based on the assumption of steady state; however, the stark blood flow effects observed in our study introduced a nonsteady state situation, which could have affected our results. Finally, a direct comparison of the depot-specific contribution to systemic lipolysis between femoral and abdominal adipose tissue was not feasible, because the measurements were not paired. However, clear differential responses appear to exist between the two depots in hypercortisolemia.

In conclusion, we have provided evidence that acute hypercortisolemia increases plasma NEFA and exerts selective effects on adipose tissue lipolysis and ATBF, supporting the concept of tissue- and depot-specific GC actions *in vivo*. Femoral adipose tissue does not appear to be a net contributor of NEFA in hypercortisolemia; however, marked changes in femoral ATBF could herald effects on femoral fatty acid trafficking that become evident only with chronic hypercortisolemia. The exact source of the excess systemic NEFA in hypercortisolemia remains elusive, but it can be speculated that it might derive from the visceral adipose tissue depot. The increase in abdominal lipolysis we observed appears insufficient to explain the marked increase in plasma NEFA during hydrocortisone infusion. Future studies are needed to address whether altered subcutaneous adipose tissue depot function and to what extent visceral adipose tissue lipolysis contributes to the pathophysiological changes in fatty acid metabolism in chronic GC excess.
