# Supplementary material for: Acute Hypercortisolemia Exerts Depot-Specific Effects on Abdominal and Femoral Adipose Tissue Function
Source: J Clin Endocrinol Metab. 2017 Feb 16;102(4):1091–101. doi: 10.1210/jc.2016-3600 (PMC5460725; doi:10.1210/jc.2016-3600)
Supplement: Supplementary file 1 [file jc.2016-3600.sd1.docx]

**Supplemental Material**

**Supplemental Methods**

**HOMA calculations**

Indices of pancreatic β-cell function and insulin resistance were calculated according to the updated homeostatic model assessment (HOMA2) using the computer model/calculator available publicly (1). The calculations on which this model is based have been described as follows:

Beta cell function (%B) = (20 × fasting plasma insulin)/(fasting plasma glucose − 3.5)

Insulin Resistance (IR) = [(fasting plasma insulin) x (fasting plasma glucose)]/22.5

Insulin Sensitivity (%S) = 1/[IR]

**Metabolic flux calculations**

*NEFA and glycerol release* was calculated as

(Venous concentration_[metabolite]_ – Arterial concentration_[metabolite]_) x ATBF

*Glucose uptake* was calculated as

(Arterial concentration_[glucose]_ – Venous concentration_[glucose]_) x ATBF

Depot-specific lipolysis independent of ATBF was calculated as the *fraction of venous and arterial NEFA* concentrations as

Venous concentration_[NEFA]_ / Arterial concentration_[NEFA]_

with the assumption that if no local net lipolysis is taking place the value would be 1.

All calculations based on the work by Frayn *et al.* (2,3).

**Supplemental Tables**

**Table 1:** Regional plasma NEFA and glycerol concentrations, calculated veno-arterial (V-A) difference. All values representing metabolite concentrations (not fluxes), mean and SEM shown. Femoral data from study 1 (n=9) and abdominal data from study 2 (n=8). Paired T-Test shown.

|  | **Control** | **Hypercortisolemia** | **p** |
| --- | --- | --- | --- |
| **Fasting femoral (0-30 min)** |  |  |  |
| NEFA arterial (µmol/L) | 563±34 | 928±59 | <0.001 |
| NEFA femoral venous (µmol/L) | 970±84 | 1183±125 | 0.177 |
| NEFA femoral V-A difference (µmol/L) | 407±72 | 255±81 | 0.240 |
| Glycerol arterial (µmol/L) | 36±2 | 49±2 | 0.005 |
| Glycerol femoral venous (µmol/L) | 139±17 | 97±17 | 0.149 |
| Glycerol femoral V-A difference (µmol/L) | 102±17 | 48±16 | 0.061 |
|  |  |  |  |
| **Postprandial femoral (200-240 min)** |  |  |  |
| NEFA arterial (µmol/L) | 19±4 | 76±13 | <0.001 |
| NEFA femoral venous (µmol/L) | 46±11 | 93±13 | 0.019 |
| NEFA femoral V-A difference (µmol/L) | 27±11 | 20±6 | 0.650 |
| Glycerol arterial (µmol/L) | 6±1 | 13±1 | 0.001 |
| Glycerol femoral venous (µmol/L) | 20±4 | 20±3 | 0.905 |
| Glycerol femoral V-A difference (µmol/L) | 15±3 | 6±2 | 0.048 |
|  |  |  |  |
| **Fasting abdominal (0-30 min)** |  |  |  |
| NEFA arterial (µmol/L) | 560±33 | 1043±79 | <0.001 |
| NEFA abdominal venous (µmol/L) | 1307±149 | 1775±106 | <0.001 |
| NEFA abdominal V-A difference (µmol/L) | 761±131 | 716±72 | 0.696 |
| Glycerol arterial (µmol/L) | 33±4 | 49±5 | 0.008 |
| Glycerol abdominal venous (µmol/L) | 180±22 | 168±18 | 0.943 |
| Glycerol abdominal V-A difference (µmol/L) | 146±21 | 129±17 | 0.485 |

**Supplemental Figures**

**Supplemental Figure 1**

**Supplemental figure 1: Systemic concentrations and adipose tissue depot-specific IL-6 release.** Systemic plasma IL-6 concentrations **(A)** during hydrocortisone (HC) or saline (S) infusion in the fasting state (n=9, study 1). Adipose tissue depot-specific IL-6 release **(B)** during infusion of hydrocortisone (black triangle femoral; black circle abdominal) or saline (black inverted triangle femoral; black square abdominal) (femoral n=9, abdominal n=4 for saline, n=7 for hydrocortisone).

**References**

**1.** **Wallace TM, Levy JC, Matthews DR**. Use and abuse of HOMA modeling. *Diabetes Care* 2004; 27:1487-1495

**2.** **Frayn KN, Coppack SW, Humphreys SM, Whyte PL**. Metabolic characteristics of human adipose tissue in vivo. *Clin Sci (Lond)* 1989; 76:509-516

**3.** **Frayn KN, Shadid S, Hamlani R, et al.** Regulation of fatty acid movement in human adipose tissue in the postabsorptive-to-postprandial transition. *Am J Physiol* 1994; 266:E308-317
